# ALKBH1 Is Dispensable for Abasic Site Cleavage during Base Excision Repair and Class Switch Recombination

**DOI:** 10.1371/journal.pone.0067403

**Published:** 2013-06-25

**Authors:** Tina A. Müller, Kefei Yu, Robert P. Hausinger, Katheryn Meek

**Affiliations:** 1 Microbiology and Molecular Genetics, Michigan State University, East Lansing, Michigan, United States of America; 2 Biochemistry and Molecular Biology, Michigan State University, East Lansing, Michigan, United States of America; 3 Pathobiology and Diagnostic Investigation, Michigan State University, East Lansing, Michigan, United States of America; Tulane University Health Sciences Center, United States of America

## Abstract

Potential roles of the abasic site lyase activity associated with AlkB homolog 1 (ALKBH1) were assessed by studies focusing on the two cellular processes that create abasic sites as intermediates: base excision repair and class switch recombination. *Alkbh1^−/−^* pups (lacking exon 3) were born at a lower than expected frequency from heterozygous parents, suggesting a reduced survival rate and non-Mendelian inheritance, and they exhibited a gender bias in favor of males (70% males and 30% females). To study ALKBH1’s potential involvement in DNA repair, fibroblasts were isolated from *Alkbh1^−/−^* mice, spontaneously immortalized and tested for resistance to DNA damaging agents. *Alkbh1^−/−^* and isogenic cells expressing hALKBH1 showed no difference in survival to the DNA damaging agents methyl-methionine sulfate or H_2_O_2_. This result indicates that ALKBH1 does not play a major role in the base excision repair pathway. To assess ALKBH1’s role in class switch recombination, splenic B cells were isolated from *Alkbh1^−/−^* and *Alkbh1^+/+^* mice and subjected to switching from IgM to IgG1. No differences were found in IgG1 switching, suggesting that Alkbh1 is not involved in class switch recombination of the immunoglobulin heavy chain during B lymphocyte activation.

## Introduction

Abasic or apurinic/apyrimidinic (AP) sites are common lesions in DNA that arise spontaneously through hydrolysis of unstable N-glycosidic bonds or are formed enzymatically through the removal of damaged bases [1]–[3]. Two cellular processes create AP sites as intermediates: base excision repair (BER) and class switch recombination (CSR), associated with DNA repair and immunological DNA rearrangement, respectively.

BER removes minor modified DNA bases that do not distort the DNA helix such as those resulting from alkylation, hydroxylation or deamination of cytosines [4]–[8]. The pathway is initiated by a damage specific mono- or bifunctional DNA glycosylase. The former cleaves the N-glycosidic bond between the damaged base and the deoxyribose backbone creating an AP site, with the DNA subsequently being hydrolyzed at the 5′-side of the lesion by AP endonuclease 1 (Ape1). The bifunctional glycosylase/lyase removes the damaged base and introduces a nick in the DNA backbone at the 3′-side of the AP site. Both types of nicks are processed and the DNA ends are religated by DNA polymerases and ligases.

CSR refers to the ability of B cells to switch antibody production from isotype IgM to IgG, IgE, and IgA by DNA recombination of the immunoglobulin heavy chain genes [9]. The current model describing this B cell-mediated process initiates with activation-induced cytidine deaminase (AID) which converts cytidines to uridines in the so-called switch (S) regions of DNA located upstream of the immunoglobulin constant region genes [10]–[14]. These uridines are recognized by the BER protein uracil DNA glycosylase (UNG), which removes the bases, leaving AP sites [15], [16]. An endonuclease then cleaves the DNA at the AP sites; when two such sites are located in close proximity on opposing DNA strands, a double-strand break (DSB) results. Creation of two DSBs leads to excision of the intervening DNA region with the breaks being repaired by non-homologous end joining (NHEJ), thereby rearranging the locus encoding the immunoglobulin heavy chain. Despite intensive efforts, no unequivocal evidence has identified the AP-endonuclease that introduces the DSB. Since AID is the only B cell specific factor required for switching, the enzymes catalyzing the other CSR-related reactions are most likely ubiquitously expressed, and several enzymes of the BER as well as the mismatch repair (MMR) pathway have been implicated in the process [17]–[19]. Because APE1 is essential for mouse embryo development, its role in CSR has been difficult to test directly. Just recently, it was shown that CSR is not completely abolished in APE1-null cells [20], suggesting that an additional, yet unidentified endonuclease could also be involved in DNA cleavage during CSR.

**Figure 1:**
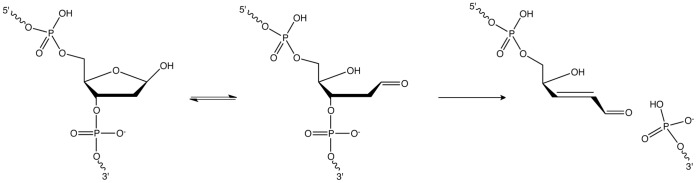
Scheme of ALKBH1’s AP lyase activity.

Mammalian AlkB homolog 1 (ALKBH1, also known as ABH1) is related to the Escherichia coli DNA repair enzyme AlkB that catalyzes the oxidative demethylation of 1-methyl adenine and 3-methyl cytosine in single-stranded (ss) DNA [21], [22]. ALKBH1 catalyzes the analogous reaction using 3-methyl cytosine, but not 1-methyl adenine, at a very slow rate [23]. It was also suggested that ALKBH1 is a methylated-histone demethylase involved in neural development by modifying the methylation status of histone H2A [24]. Human ALKBH1 (hALKBH1) exhibits an additional activity as an AP lyase (Fig. 1), a reaction of unknown in vivo role [25]. Because ALKBH1 is ubiquitously expressed in different tissues and highly expressed in the spleen and lymphoblasts [26], [27], we hypothesized that ALKBH1’s AP lyase activity could play a role in either BER or CSR.

To test this hypothesis, Alkbh1^−/−^ mice [28] were recreated from *Alkbh1^+/flox^* animals and characterized in terms of their overt phenotypes. Fibroblast cells were isolated from these animals, immortalized, and then analyzed for their resistance to DNA damaging agents to examine a possible involvement of ALKBH1 in BER. To study the potential role of ALKBH1 in CSR, splenic B cells were isolated from Alkbh1^−/−^ and wild type mice and stimulated *in vitro* to induce CSR from IgM to IgG1.

## Results

### Phenotypic Characterization of Exon 3-Deleted Alkbh1^−/−^ Mice

**Figure 2:**
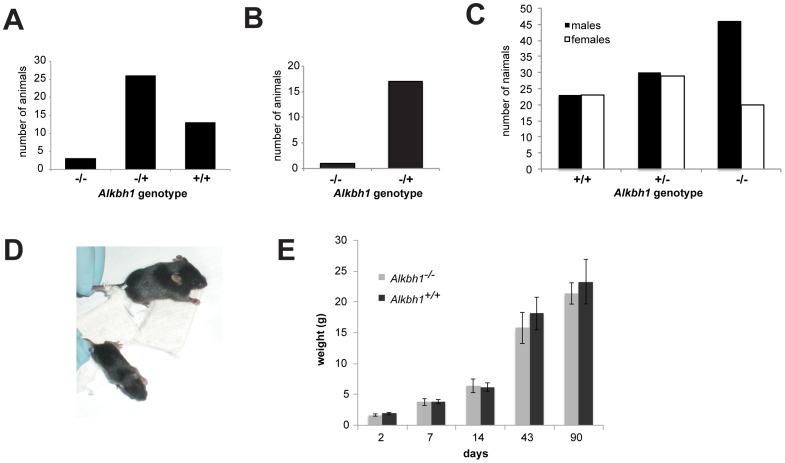
Characterization of ALKBH1 deficient mice. A. Offspring distribution of crosses between heterozygous male and female mice. Numbers of pups from nine different litters are shown, demonstrating non-Mendelian inheritance of the knockout allele. B. Offspring distribution after crosses between Alkbh1^+/−^ females and Alkbh1^−/−^ males. C. Male-to-female ratios of Alkbh1^+/+^, Alkbh1^+/−^, and Alkbh1^−/−^ mice show a distorted sex ratio in favor of males for the knockout mice. D. Different sizes of Alkbh1^−/−^ and wild type (larger pup) littermates at two weeks of age. E. Weight development of Alkbh1^−/−^ and wild type pups. Alkbh1^−/−^ and wild type pups were derived from breeding either two ALKBH1 deficient or two wild type parent animals. For each time point, the weights of at least twelve animals were used to calculate the average and the error bars represent standard deviations.

Two distinct types of mice lacking ALKBH1 are described in the literature. An Alkbh1^−/−^ mouse described by Pan et al. [28] lacks exon 3, whereas Nordstrand and coworkers [29] deleted exon 6 of the gene. Both deletions result in a null allele as shown by Western blot or RT-PCR amplification analyses. The two studies of ALKBH1 deficient mice reported on different physiological and developmental features of these animals. For the investigations described here, frozen Alkbh1^fl/+^ embryos were obtained from Dr. S. M. Lipkin (University of California - Irvine) and Alkbh1 exon 3 knockout mice were recreated in a C57/BL6 background. Breeding studies showed that these Alkbh1^−/−^ mice are born at a lower than expected frequency from heterozygous parents, suggesting a reduced survival rate and non-Mendelian inheritance (Fig. 2A, p value = 0.028 in the χ^2^-test). The same observation was made when breeding heterozygous females with homozygous *Alkbh1^−/−^* males (Fig. 2B, p value = 0.00016, χ^2^-test). ALKBH1 deficient mice display a significant gender bias, with more males than females being born (Fig. 2C, p value = 0.0036 in the χ^2^-test). Furthermore, Alkbh1^−/−^ animals are smaller than their wild-type and heterozygous littermates when present in mixed litters (Fig. 2D). These observations are in agreement with the extensive study of the exon 6 deletion strain examined by Nordstrand and coworkers [29]. However, in litters with only ALKBH1 deficient pups (born from Alkbh1^−/−^ parents), no differences in birth weight or growth rate to wild-type pups are observed (Fig. 2E); a phenotype which had not been documented before. This finding is consistent with Pan and coworkers’ conclusion of a defect in placental trophoblast function, and suggests that the ALKBH1 deficient fetus cannot compete *in utero* with wild-type littermates.

### Cellular Sensitivity of Alkbh1^−/−^ and hALKBH1 Expressing Cells to H_2_O_2_ and Methyl Methanesulfonate

**Figure 3:**
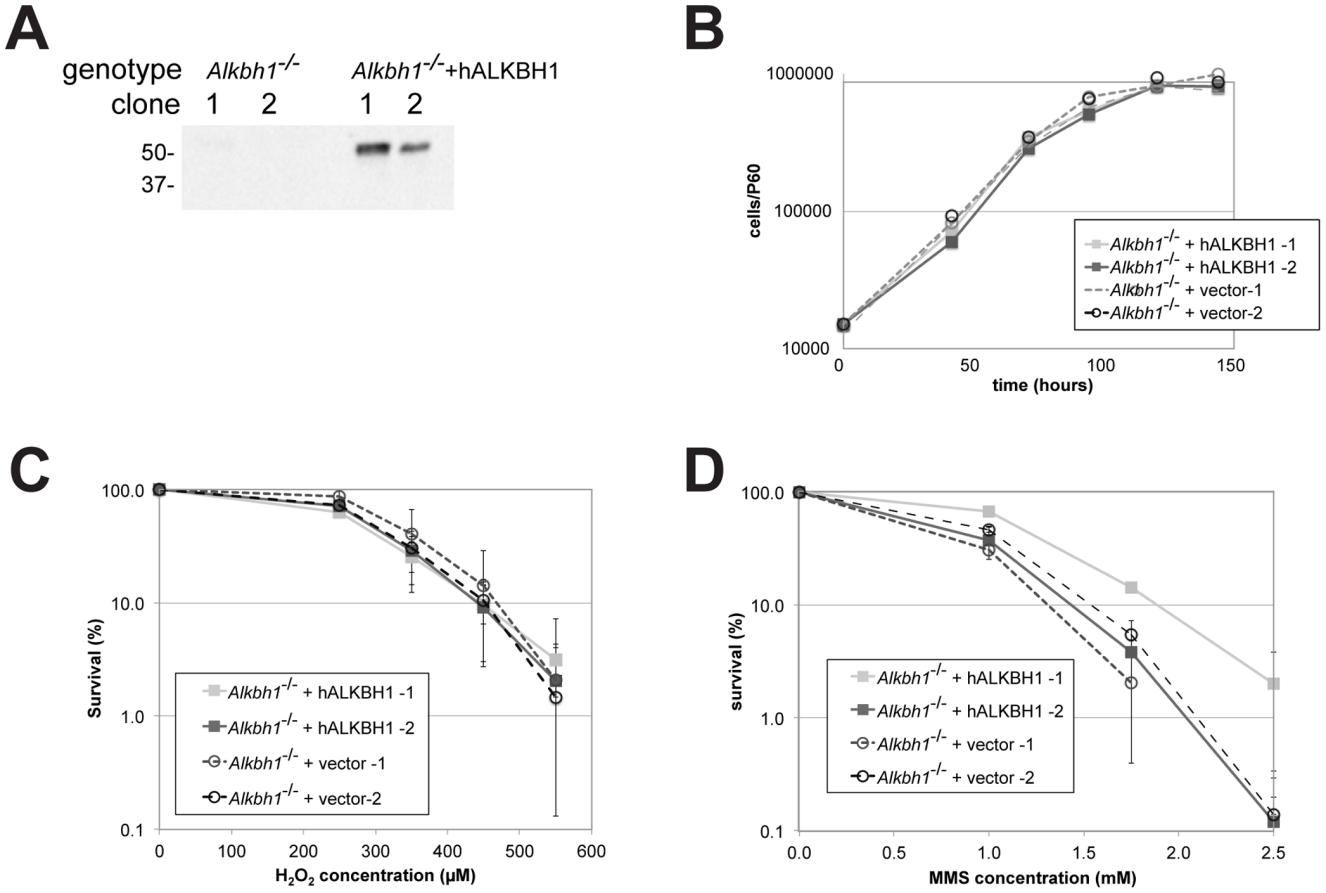
ALKBH1 deficient fibroblasts are not more sensitive to DNA modifying agents than hALKBH1 expressing cells. A. Expression of hALKBH1 in mouse Alkbh1^−/−^ fibroblasts. Lysates of indicated clones (20 µg) were analyzed by Western blot using a monoclonal anti-ALKBH1 antibody. The positions of two molecular mass markers are shown in kDa. B. Growth curves of Alkbh1^−/−^ fibroblasts and the same cells producing hALKBH1. Cells were plated at low cell density, harvested at the indicated time point by trypsinization and counted. Survival curves for Alkbh1^−/−^ fibroblasts expressing hALKBH1 or containing a vector control when treated with various concentrations of H_2_O_2_ (C) or MMS (D). Error bars indicate standard deviations derived from three (H_2_O_2_) or two (MMS) independent experiments.

To assess the function of ALKBH1 in BER, ALKBH1 deficient fibroblasts were derived and then complemented with human ALKBH1. Spontaneously immortalized fibroblast cells were isolated from the peritoneal wall of an Alkbh1^−/−^ mouse. These cell lines were infected with recombinant retroviruses expressing hALKBH1 or control virus made from empty vector and single clones derived from infected cells were isolated. Expression of human ALKBH1 was demonstrated by Western blot analysis (Fig. 3A). Cell growth rates indicate that ALKBH1 deficient cells grow as well as cells expressing human ALKBH1 (Fig. 3B).

We next examined the effects of DNA damaging agents on these isogenic cell strain. Two DNA damaging agents that induce small modifications of the DNA bases were chosen. H_2_O_2_ oxidizes DNA bases and methyl methanesulfonate (MMS) introduces alkylation; both lesions are known to be repaired by BER [1]. To test the effect of these agents on cell survival, two clones deficient in ALKBH1 and two expressing hALKBH1 transgene were exposed to the chemicals at different concentrations, plated at identical clonal densities, and colonies were counted after 6 days. The clones lacking ALBKH1 showed no statistical difference in their survival to H_2_O_2_ and MMS compared to those expressing the human homolog, indicating that ALKBH1 is not required for repair of either H_2_O_2_ and MMS induced DNA damages. This result suggests that ALKBH1 is not essential for BER (Fig. 3C and D).

### Testing the Hypothesis that ALKBH1 Functions in CSR

Because ALKBH1 is highly expressed in spleen and lymphoblasts [26], [27], we considered the possibility that ALKBH1 might have a function in the immune system, such as an AP endonucleolytic activity during CSR.

**Figure 4:**
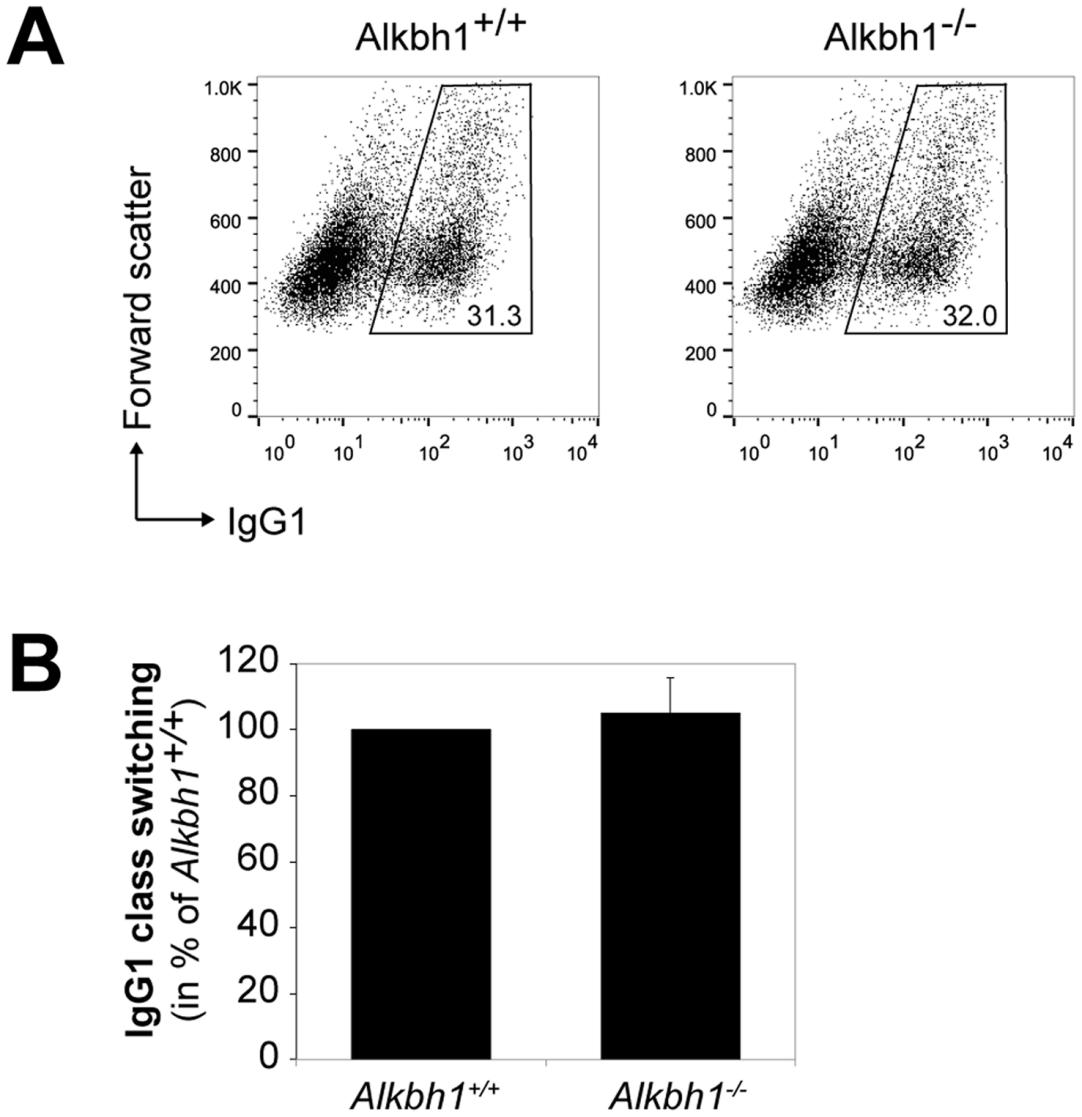
Ig class switching to IgG1 is not reduced in Alkbh1^−/−^ splenic B cells. A. Flow cytometry results showing surface antibody expression four days after treatment to switch to IgG1. B. Analysis of flow cytometry data related to formation of IgG1 in ALKBH1 deficient splenic B cells normalized to the switching rate of Alkbh1^+/+^ B cells. Four mice were used for each genotype and error bars represent standard deviations from these four independent experiments.

To test this hypothesis, splenic B cells were isolated from 8–10 week old male Alkbh1^−/−^ and male Alkbh1^+/+^ mice, and both cell populations were activated to undergo CSR in culture. Since cell proliferation rates can affect switching rates, the growth rates of the two cell types were monitored by trypan blue staining. Alkbh1^−/−^ B cells exhibited slightly greater growth than Alkbh1^+/+^ cells after 4 d, but the difference was not statistically significant (data not shown). Flow cytometry analysis showed no significant difference in CSR efficiency for generating IgG1 when comparing Alkbh1^+/+^ and Alkbh1^−/−^ B cells (Fig. 4). In a single assay, we also examined CSR to make IgG2a for these two genotypes and found no significant difference confirming the results obtained with switching to IgG1 (data not shown). We conclude that ALKBH1 does not play a role in Ig CSR.

## Discussion

Two Alkbh1^−/−^ mouse models have been created by different approaches [28], [29] and used to define distinct types of phenotypes. Pan and coworkers, studying an exon 3 deletion, found intra-uterine growth retardation in Alkbh1^−/−^ mice and suggested that ALKBH1 is involved in placental trophoblast lineage differentiation and transcriptional regulation [28]. In contrast, Nordstrand et al. created an exon 6 deletion and reported on non-Mendelian inheritance, a distorted gender bias in favor of males, and effects on embryonic development [29]. Both deletions produced null animals with regard to the 2-oxoglutarate dioxygenase domain of ALKBH1; however, an isoform of unknown function reported in the NCBI database (EAW81302) and consisting of only the first 185 amino acids would be encoded by the exon 6 knockout, but not by that lacking exon 3. This truncated form of ALKBH1 does not contain the iron-binding ligands, consistent with ALKBH1 having an additional role independent of its DNA or histone demethylating abilities. The results obtained here using the strain deleted in exon 3 clearly demonstrate that the two types of ALKBH1 deficient mice have similar phenotypes with regard to non-Mendelian inheritance, growth retardation, and distorted sex ratio. Furthermore, no additional overt phenotype was associated with the inability to synthesize the putative isoform when using the exon 3 deletion mutant. As reported before, Alkbh1^−/−^ pups exhibited smaller birth weights when born in heterozygous litters [28], [29]. Interestingly, the weights of Alkbh1^−/−^ pups derived from homozygous ALKBH1 deficient parents did not differ from those of pups from wild type litters. This observation suggests that wild type embryos have an advantage over Alkbh1^−/−^ embryos in the uterus, but when only ALKBH1 deficient embryos are developing, no intra-uterine growth retardation is observed. This phenomenon may be due to the reduced litter size of Alkbh1^−/−^ female mice compared to wild type mice (3 pups/litter versus 5.17 pups/litter for six breedings each; [29]). Moreover, this result is consistent with Pan *et al.*’s conclusion that *Alkbh1^−/−^* mice have a defect in placental trophoblast function [28]. Although intra-uterine growth retardation is not strongly associated with defects in BER, it has been linked with deficits in DNA repair pathways [30], [31]. It is possible that the rapid replication of placental trophoblasts requires efficient BER with a unique requirement for ALKBH1. In the absence of ALKBH1, repair might be less efficient, thereby impeding cell growth and potentially explaining why Alkbh1^−/−^ pups are only growth retarded when they do not compete for uterine space with ALKBH1 proficient embryos.

Assessing possible in vivo roles for ALKBH1’s in vitro AP lyase activity [25], we hypothesized that it might be involved in the BER pathway. Several mono- or bifunctional glycosylases with different substrate specificities remove damaged DNA bases and, if not associated with a lyase activity, an endonuclease introduces a DNA break. Animals or cells deficient in one of the enzymes involved in the early steps of BER show a broad spectrum of phenotypes; mice deficient in some of these enzymes have the expected defects in DNA repair, while others lack obvious DNA repair deficiencies but have other phenotypic abnormalities. For instance, Ape1^+/−^ mice show reduced repair capacity and Ape1 deficiency is reported to be lethal [32], [33]. Similarly, Polβ deficient cells are hypersensitive to methylating agents due to the missing lyase activity of the enzyme [34]. Mice lacking the DNA glycosylase NEIL1, which acts on oxidized pyrimidines, show reduced DNA repair capacity only in mitochondria, but exhibit symptoms reminiscent of human metabolic syndrome such as severe obesity, dislipidemia and fatty liver disease [35]. In contrast to these phenotypes, Alkbh1^−/−^ mice have a novel phenotype, exhibiting a distorted sex-ratio, non-Mendelian inheritance, and intra-uterine growth retardation. These phenotypes do not appear to be related to a defect in BER; however, mice lacking a BER protein do not always show an overt phenotype and a more detailed analysis of the mutations or damage to the genome is needed. For instance, OGG1 deficient mice appear healthy and are indistinguishable from their wild-type littermates. Only over periods of time do oxidized guanines accumulate in certain tissues [36]–[38]. We therefore extended our analysis and compared fibroblasts lacking ALKBH1 with hALKBH1 expressing cells with respect to their survival rates to DNA damaging agents. Alkbh1^−/−^ fibroblasts did not show any hypersensitivity to the two chemicals H_2_O_2_ and MMS that introduce small DNA base modifications. This finding suggests that mammalian ALKBH1 is most likely not involved in BER. However, we cannot rule out that its activity is secondary to other endonucleases in the cell as was shown for the bifunctional glycosylase Nth acting on thymine glycol lesions. Mice lacking the nth gene do not show an overt phenotype and embryonic cells were as sensitive to H_2_O_2_ or menadione as wild-type cells confirming the presence of other enzymes acting on the same substrate [39].

We next tested a possible role of ALKBH1’s AP lyase activity in CSR. The enzyme’s preference for double-stranded oligonucleotides with two AP sites in close proximity over substrates with only one AP site, the low turnover number and tight binding to its product [25], and the expression profile in spleen and lymphoblasts [26], [27] makes this protein a reasonable candidate for the DSB-introducing endonuclease that functions in DNA S regions during CSR.

Several endonucleases associated with the BER, MMR, and NHEJ DNA repair pathways have been suggested to play a role in CSR. One key example is Ape1, the main mammalian AP endonuclease of the BER pathway. The switching rate in heterozygous mice was only reduced to 77% of the wild type cells in CSR [40]. In the same study, an Ape2^y/−^ mutant was examined and shown to have a greater reduction in its switch rate (to 65%) than Ape1^+/−^. Surprisingly, the double mutant did not exhibit further reduction in switching (64%), indicating the need for another endonuclease to catalyze DSBs. Other DNA repair endonucleases are important for the conversion of single-strand breaks into DSB. B cells isolated from mice deficient in the endonuclease activity of Pms2, a component of the MLH complex in MMR, showed a 40–60% reduction in switching compared to wild type cells [41]. The exo- and endonuclease Mre11 of the MRN complex involved in NHEJ might also play a role in switching on the basis of its AID-dependent localization to nuclear foci at the Ig heavy chain locus [42]. Dinkelmann and coworkers created mice with conditional B cell Mre11^−/−^ leading to the elimination of the endo- and exonuclease activity, but not the MRN complex [43]; B cells isolated from these animals exhibited a 50% lower switching rate than cells from control mice. Significantly, none of the above mice showed an absolute requirement for any one endonuclease in order to carry out CSR indicating that several different endonucleases may be involved, perhaps with “backup” enzymes capable of substituting for the main endonuclease when it has been deleted. However, switching assays with splenic B cells isolated from Alkbh1^+/+^ and Alkbh1^−/−^ mice showed no difference in the switching rate to IgG1 or IgG2a. This result suggests that ALKBH1’s AP lyase activity is not involved in the process of genetic rearrangement in B cells, although we cannot rule out that several glycosylases and AP endonucleases have redundant functions that might mask a possible effect.

## Materials and Methods

### Mice

Frozen embryos of Alkbh1^+/flox^ mice [28], kindly provided by Dr. Lipkin, were implanted into C57/BL6 mice at the Transgenic Animal Model Core of the University of Michigan. The Alkbh1^−/−^ mice used in this study were created by breeding Alkbh1^+/flox^ female C57/BL6 mice to E2A Cre male mice (Jackson Laboratory). The progeny mice were then intercrossed to generate *Alkbh1^−/−^* offspring. All mice were analyzed for their genotype by PCR amplification using forward primer all_Alkbh1_oligo (5′-ccaggcactagttcttccatgagcaac-3′) and reverse primers Alkbh1_wt_oligo (5′-aggactgtgggaacagagcaaagaggtc-3′), Alkbh1_flox_oligo (5′-ctctagtaagtataggaacttcggatcc-3′) and Alkbh1_Cre-del_oligo (5′-tcctggaactcactctgtagaccaag-3′), respectively [28], as well as one additional reverse primer Alkbh1_del2_oligo (5′-gcccggcgaaccttcagttctgagtcgacg-3′) to verify the deleted allele. Mice were housed in an Institutional Animal Care and Use Committee-approved facility at Michigan State University. All experiments were approved by the Michigan State University Animal Care and Use Committee.

### Creation of Immortalized Alkbh1^−/−^ and Human ALKBH1-expressing Fibroblasts

Immortalized fibroblasts from Alkbh1^−/−^ mice were established using peritoneal walls isolated from the respective animals. The peritoneal wall was harvested, washed with phosphate buffered saline and cut into small pieces. The cells were transferred to tissue culture dishes and grown in Dulbecco’s Modified Eagle Medium (DMEM; Invitrogen) supplemented with 10% fetal bovine serum (Atlanta Biologicals) and 1x penicillin-streptomycin solution at 37°C with 5% CO_2_ until small patches formed. These were transferred separately to new tissue culture dishes and grown as before. Cultures were grown until the cells spontaneously immortalized. To create hALKBH1 expressing fibroblasts, hALKBH1 was PCR amplified from pBAR67 [44] with primers 5′- ggaattccgcgagatggggaagatgg-3′ and 5′-gttagcagccggatcctcgaggtctcagc-3′ introducing restriction sites EcoRI and XhoI, respectively, and cloned into pGEM-T Easy to verify the sequence. The insert was subcloned into pMSCVneo (Clontech) using the same restriction sites, creating plasmid pABH44. To produce retrovirus, human embryonic kidney 293T cells at about 70% confluency were transfected with pABH44 and the helper plasmids pcPG and pVSVG using fugene6 according to the manufacturer’s instructions (Roche) and incubated for 24 h at 37°C with 5% CO_2_. Subconfluent Alkbh1^−/−^ fibroblasts were exposed to the virus for 2.5 h at 37°C and 5% CO_2_ and selection (500 µg G148/ml; Invitrogen) was applied after overnight incubation. G418 resistant colonies were transferred to 24-well plates and further propagated. Once clones were established, cell lysates were made and product of hALKBH1 was verified by Western blot. To generate a control strain, Alkbh1^−/−^ cells were treated with virus containing vector only and clones were generated identically.

### Growth Curves

Alkbh1^−/−^ and Alkbh1^−/−^ expressing hALKBH1 fibroblasts were plated at 3000 cells/60-mm diameter dish and grown at 37°C in a humidified incubator with 5% CO_2_. At specific time points, two or three dishes were harvested by trypsinization and the cells centrifuged for 5 min at 2000 rpm. The pellet was resuspended in 0.5–1 ml DMEM complete and the cells were counted using a Countess Automated cell counter (Invitrogen).

### DNA Damage Survival Assays

Cells from confluent tissue culture dishes were seeded at 200,000 cells per 100-mm diameter dish and grown for 24 h. All cultures were harvested by trypsinization, centrifuged at 2000 rpm for 10 min and resuspended in DMEM containing 5% bovine calf serum and 0.5x penicillin-streptomycin solution. To expose the cells to H_2_O_2_ and MMS, 7000 cells were incubated in solution in DMEM (without serum and antibiotics) for 1 h at the respective concentrations and shaken regularly. After exposure, about 280 cells were plated per 60-mm diameter dish and the colonies were counted after six days of incubation at 37°C and 5% CO_2_.

### B Cell Isolation

Mice were sacrificed by CO_2_ asphyxiation and their spleens were harvested. Splenocytes were obtained and splenic B cells were isolated by using a B cell isolation kit (Miltenyi). All steps were carried out according to the manufacturer’s protocols.

### B Cell Switching Assays

Isolated B cells were stimulated with 20 µg/ml of lipopolysaccharide (LPS, Sigma) and 5 ng/ml of IL-4 (R&D Systems) to switch to IgG1 and with 20 µg/ml of LPS and 10 U/ml of interferon-γ to form IgG2a. After 4 days, the stimulated cells were stained with fluorescein-isothiocyanate (FITC)-labeled rat anti-mouse IgG1 (PD Pharmingen) or goat F(ab’)_2_ anti-mouse IgG2a (Southern Biotech), respectively, and analyzed by flow cytometry.
